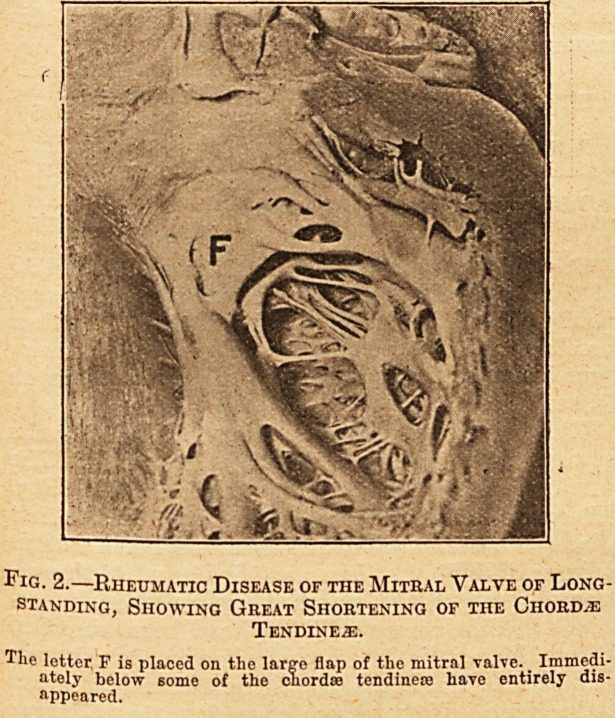# Some Affections of the Heart Connected with Sudden Death

**Published:** 1907-02-16

**Authors:** Theodore Fisher


### Hospital Clinics.

### SOME AFFECTIONS OF THE HEART CONNECTED WITH SUDDEN DEATH

By THEODORE FISHER, M.D., M.R.C.P.

Medical men are often called upon to investigate the cause of death in some person who has fallen dead in the street, or has died suddenly when engaged in some quiet occupation at home, or possibly has been found dead in bed. If we are to believe the reports of inquests published in the newspapers, there appears occasionally to be considerable uncertainty in the mind of the medical witness as to the exact cause of death.

In making such a remark it is not intended to convey the idea that sympathy is felt with those who maintain that all post-mortem examinations requiring public inquiry should be made by a medical man who has had special experience of post-mortem work. Although I have been present at about 2,500 autopsies, such experience would not by any means enable me to assign definitely the cause of death in every case apart from some knowledge of signs or symptoms of disease which may have existed during life. While, however, I feel that evidence should be given regarding the presence or absence of signs of disease during life, it seems to me also that many medical men who are required by law to ascertain the cause of death from post-mortem appearances, frequently need a more accurate knowledge of these appearances. Such an opinion is not expressed without having come into contact with those who give evidence as to the cause of death. In hospitals it is the medically qualified residents who are the expert witnesses in coroner's courts. They are generally fresh from some large medical school, are well trained in nearly every branch of medicine and surgery, yet their ability to recognise appearances of simple character in connection with morbid anatomy is often lacking.

When we speak of sudden death, both medical men and those without medical training, generally think at once of the heart. And it is needless to say that they are right to regard it as all-important to the maintenance of life. In passing, it may be remarked that formerly, in reports of inquests in the daily papers—and occasionally I think it is so still—apoplexy is mentioned as a cause of sudden death. Absolutely sudden death from such a cause, however, is extremely rare. It is necessary to remember that cardiac action is virtually independent of the brain. The emotions of the mind acting through the brain may disturb the heart's rhythm, and such impulses may temporarily, or in very rare instances even permanently, arrest cardiac action. Yet the brain itself is not responsible for the steady continuance of cardiac systole and diastole, as it is for the respiratory movement of the chest; and consequently if an acute lesion of the brain is to produce sudden death, it must either cause it by shock or by injuring the respiratory centre. Should respiration cease, cardiac action, it is needless to say, will soon also cease from want of aëration of the blood which flows through the coronary arteries.

The most common causes of arrest of cardiac action arise not without the heart, but within it. In connection with these causes arising within the heart, however, there are possible fallacies. Perhaps the most common is the importance attached to what is called fatty degeneration of the heart. Again, although the words "mitral regurgitation" are not often used in coroner's courts, this morbid condition is another stumbling-block. Mitral regurgitation, it is true, is not considered to be a common cause of sudden death, yet the importance of regurgitation through the mitral orifice, as a disease, is deeply ingrained in the minds of most of us; and this mental attitude affects the way those of little experience examine a heart in which they expect to find the cause of death. There being no fatty degeneration of the cardiac muscle that they can detect, and no disease of the aortic valve, they examine the cusps of the mitral valve, and almost invariably succeed in discovering something abnormal which they consider sufficient to account for death. The most common error will be referred to later, but it may not be out of place briefly to refer to the size of the mitral orifice. Some students appear to be taught that the mitral orifice normally admits the tips of two fingers. It is scarcely necessary to remark that the tips of fingers differ much in size; yet when we consider how the height and weight of the human body varies, the size of the normal mitral orifice is remarkably constant. It ranges only very slightly above and below 4 inches in circumference. Two of my own finger-tips measure about 3½ inches, whereas three finger-tips measure exactly 4 inches. Three finger-tips are frequently stated—and it seems to me are more correctly stated than two—to be a gauge of the size of a normal mitral orifice.

Probably, however, few of those inexperienced in post-mortem work lay stress upon evidence of dilatation of the mitral orifice as measured by the finger-tips. There is a much more common source of error. This error is the discovery of a thickened edge to the mitral valve. The expression "edge of mitral valve thickened" is a familiar one, and the reason why such thickening is so often thought to be present and to be the consequence of disease is because one feature of the anatomy of the mitral valve is not sufficiently borne in mind. The cusps of the mitral valve are composed of layers of endocardium covering strands of fibrous tissue, and the point to which I wish to draw attention is that these fibrous strands are prolongations of the chordæ tendineæ. The chordæ tendineæ, on becoming inserted into the flaps of the mitral valve, become minutely subdivided, and their fibres interlace in their course towards the mitral ring at the base of the flaps. The interlacing of these fibres occurs chiefly just above the margin of the valve, and in the situation of this interlacing there is a band of thickening. During the process of examination of the heart this thickening is often made to appear greater than it really is. In examination of the heart, in order to obtain a good view of the flaps of the mitral valve, not only is the left ventricle laid open, but the mitral orifice is divided between the flaps, without, however, severing the connections of the chordæ tendineæ with the muscular pillars. Whether the heart is then held in the hand or is placed on the post-mortem table, abnormal tension is placed on some of the chordæ tendineæ, and this tension causes puckering of the edge of the large flap of the mitral valve. Let this flap, however, be held up against the light, that is so that the light is seen through it, and all the apparent thickening and abnormal puckering will resolve themselves into the normal structure of the valve. The course of the divided fibres of the chordæ tendineæ will then be clearly seen, and the part the fibres play in producing the thickening which is normally present be made evident.

Having indicated a mistake that is to be avoided, we may next consider in what way inflammation affects the mitral valve. The early evidences of inflammation, it is needless to remark, are small vegetations over the position of contact of the edges of the flaps. These are, however, in cases of rheumatic endocarditis of no importance in themselves. If inflammation were limited to the site of the vegetations, endocarditis would not be followed by serious consequences. Unfortunately the inflammation extends far more widely in too many instances, and does not end with the attack of rheumatic fever in the course of which it originated. The inflammation generally affects the fibrous structures of the chordæ tendineæ from their origin at the apices of the musculi papillares to their insertion into the ring at the base of the flaps of the mitral valve. This inflammation is also often progressive. The continued movement of the valve probably acts as a source of irritation to the formation of new fibrous tissue. This fibrous tissue contracts, but does not produce puckering of the mitral valve. Puckering is a sequel of irregular inflammation. The inflammation which succeeds rheumatic endocarditis spreads over the whole of the mitral flaps and along the chordæ tendineæ connected with them. Inflammation of more irregular character may occur in association with infective endocarditis. This form of endocarditis is not by any means always acute; it is sometimes of long duration, and may heal in one place while it spreads in another. As a consequence of such local healing, I have seen very definite puckering produced. But to return to rheumatic endocarditis, the newly-formed fibrous tissue in the chordæ tendineæ and in the valve contracts, and contracts uniformly, with the resulting effect that the edges of the flaps are drawn downwards towards the apex of the ventricle and inwards towards one another.

The difference in length and thickness of the chordæ tendineæ in the early and late stages of rheumatic endocarditis will be made evident by a reference to the accompanying photographs. Fig. 1 shows rheumatic vegetations, which, it may be remarked in passing, are unusually large for vegetations of rheumatic origin; but the

Fig. 1.—Early Rheumatic Disease of the Mitral Valve Showing the Chordæ Tendineæ Free from Thickening.

The letter F is placed just above the line of attachment of the lesser flap of the mitral valve, and points by a dotted line to the larger flap. The letter C is placed on chordæ tendineæ, passing to the lesser flap, and the letter T is situated on the chordæ tendineæ attached to half of the margin of the larger flap.

chordæ tendineæ are little, if in any degree, thickened. Fig. 2 shows the large flap of the mitral valve drawn down until it is in contact with one of the muscular pillars, and the contraction of fibrous tissue which has produced this has caused the disappearance of some of the chordæ tendineæ. These features of inflammation of the mitral valve are apparently not widely recognised, and it seems that teachers of medicine often speak as if unaware of the important part played by the chordæ tendineæ in disease of the mitral valve. It is not too much to say that *rheumatic disease of the mitral valve is mainly a disease of the chordæ tendineæ*. It has previously been mentioned that the contraction of the chordæ tendineæ draws the flaps of the mitral valve downwards and inwards towards one another—in other words, it produces a narrowing of the orifice, or what we know as mitral stenosis. The flaps are, however, generally prevented from closing efficiently, and regurgitation through the orifice is also therefore present. During life the regurgitant murmur alone may attract attention, where after death the most important condition found proves to be mitral stenosis. We may with safety make another statement, and say that when in a case of sudden death *there is no evidence of stenosis of the mitral orifice, any abnormality of the mitral orifice that may be found has probably had little or nothing to do with the cause of death*. Although such a strong statement may not be found elsewhere, yet it can be made with considerable assurance, and I hope its apparent unreasonableness will disappear when we deal with disease of the cardiac muscle. It is necessary, however, to add that uncomplicated mitral stenosis is a very rare cause of sudden death.

Although sudden death as a consequence of uncomplicated mitral regurgitation may be said not to exist, there are exceptions or, at least, apparent exceptions. It has fallen to my lot to make a post-mortem examination upon one case, and one case only, where it appeared that mitral regurgitation due to some disease of the mitral valve was the cause of sudden death. The case to which I refer occurred in a young man whom I happened to meet during life under what must be considered, in the light of what happened after, somewhat curious circumstances. He called one afternoon in a cab, asking whether, since the medical attendant of the family was out, I would go to see a girl who had attended hospital as an out-patient under my care, and had, on the afternoon of his call, been taken suddenly ill. I went, but on arrival found the girl was dead. The young fellow, I learned, was engaged to be married to the girl, and the shock of the news of her death brought on, soon after our arrival, an attack of dyspnœa. It appeared that he had suffered from rheumatic fever, which, his medical attendant had told him, had affected his heart. A few months later the body of a young man, who had died suddenly when running to catch a tramcar, was on the post-mortem table. I at once recognised the corpse as that of the young man who had called to fetch me to see his dying fiancée. On examining his heart little evidence of disease was found. There was nothing abnormal visible to the naked eye in the cardiac muscle; the segments of the aortic and mitral valves were healthy; and although some thickening of the chordæ tendineæ and of the flaps

FIG. 2.—RHEUMATIC DISEASE OF THE MITRAL VALVE OF LONG-STANDING, SHOWING GREAT SHORTENING OF THE CHORDÆ TENDINEÆ.

The letter F is placed on the large flap of the mitral valve. Immediately below some of the chordæ tendineæ have entirely disappeared.

of the valve existed, and some regurgitation had no doubt been present, there was nothing that could have interfered seriously with the mechanical action of the heart.

It seems to me that rheumatism not uncommonly interferes with the nutrition of the cardiac muscle without producing abnormal appearances that can be recognised by the naked eye or even sometimes by the microscope. Occasionally, it may be remarked, such naked-eye or microscopical lesions are very evident, but there are other cases in which it appears that the cardiac muscle must have been at fault, where no very definite evidence of disease can be detected. In such a case as that just related, where there was only trifling valvular disease, it seems reasonable to conclude that the muscular wall of the heart must have been weakened in a way that rendered it liable to sudden arrest of action during strain. Some people who have had rheumatism, although no murmurs indicative of valvular disease are audible, are liable to attacks of dyspnœa, which may be accompanied by pain. In the subjects of these attacks a sudden fright or a slighter cause, such as some annoyance, may start the dyspnœa. This association of dyspnœic attacks with a definite exciting cause may lead the friends or medical attendant to consider the outbursts to be nothing but hysteria. It will have been noticed, however, that shock brought on an attack of dyspnœa in the above-mentioned young man, and that something more than hysteria was present is evident in that he died suddenly. Yet, although in his case there was a fatal ending, I do not think attacks of dyspnœa following rheumatism, at least when there is no evidence of valvular disease, are usually of serious import; but in order to express a very definite opinion upon such a point, cases would need to have been watched for many years. In connection with the above case it may be interesting to mention that it is within the bounds of possibility that the nervous shock weakened the heart. It seems to me that shock can definitely weaken the heart for a considerable time. Several cases of this nature have come under my notice.

In one instance a girl, aged 21, was introduced by a lodging-house keeper, either through some strange misunderstanding or from astonishing and callous thoughtlessness, into a bedroom where the young man to whom she was engaged was lying dead. The girl knew he was ill, but apparently, when she entered the room, was not even aware that he had been in any great danger. The sudden shock of learning the truth in this cruel manner produced so much disturbance of the heart that she was unable to leave the house for two hours, at the end of which time, by the aid of a friend, she was taken home. The following day, dyspnœa and cardiac pain returned. When she came under my notice, several months later, she was still subject to these attacks, and, when walking fast or going upstairs, she suffered from breathlessness. I have met with similar cases following such incidents as the presence of burglars in a house, the jumping of a large dog on to the patient's back, and after seeing a relative fall down in an epileptic fit. In one of these cases swelling of the legs was associated with the symptoms of cardiac weakness. This seems to show that, in this case at least, there was something more than a neurosis. It is easy to consider any morbid condition that appears obscure to be purely functional, and although it may be tempting to take this view of weakness of the heart following fright, it seems to me to be possible that unusually strong impulses, such as must occur in exceptional conditions of nervous shock, may affect the nutrition of the heart, just as some progressive lesions of the central nervous system appear occasionally to originate in a peripheral injury. It is interesting to note in this connection that there are recorded cases of death some days after a severe shock has been received. A pathetic instance is the death of Vanessa, who is said to have died a week after Swift had angrily, in person, returned the letter she had written to Stella.

We are, however, wandering somewhat from the question of sudden death associated with lesions of the mitral valve. It has been previously mentioned that sudden death associated with definite disease of the valve is rare, and that where such death occurs it does not necessarily follow that the disease of the valve has been mainly responsible for the fatal issue. We have only lightly touched upon the importance of the cardiac muscle, but it may be mentioned here that fibroid patches, possibly as large as the little finger-nail, are sometimes found in cases of death from mitral stenosis. Cases of death by slow cardiac failure are now referred to; but it is in cases in which such patches are present that sudden death would be most likely to occur. I do not happen to have had to investigate a case of sudden death from mitral stenosis, so that my own personal experience can only indicate probabilities. Something further will need to be said about the mitral orifice when sudden death associated with affections of the cardiac muscle is considered; but in closing these remarks upon disease of the cusps of the valve, it may be interesting to give an illustration of the apparently small inconvenience a serious lesion of the mitral valve may occasion even to a strenuous life. In a man, aged 65, who had died of bronchitis, I found a mitral valve greatly thickened, and so stenosed that it only admitted the tip of one finger. On inquiry it was ascertained that the man had suffered from five attacks of rheumatic fever during the course of his life, yet apparently was unconscious of anything having been wrong with his heart. He had lived an active life as a horse trainer until within a few weeks of his death. It is needless to remark, however, that instances of this character are not frequently met with. The majority of those affected with mitral stenosis die between the ages of 30 and 40 years.

## Figures and Tables

**Fig. 1. f1:**
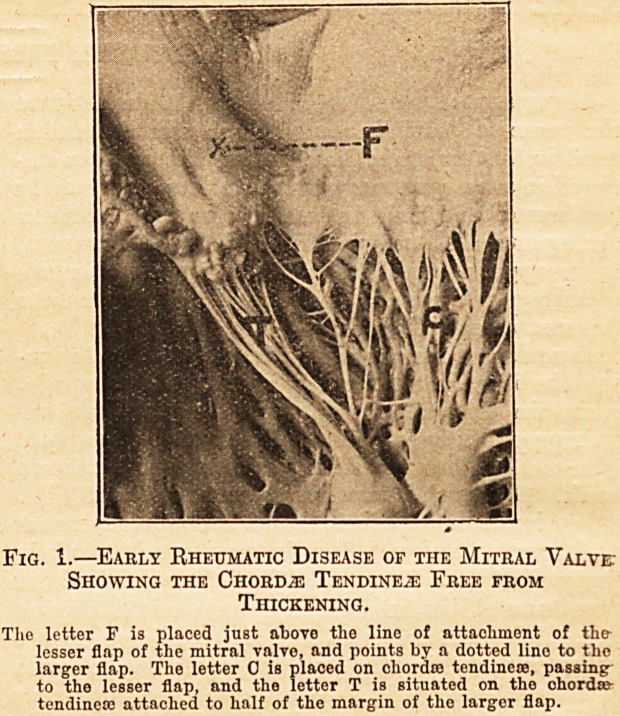


**Fig. 2. f2:**